# Human Melanoma-Derived Extracellular Vesicles Regulate Dendritic Cell Maturation

**DOI:** 10.3389/fimmu.2017.00358

**Published:** 2017-03-29

**Authors:** Rachel L. G. Maus, James W. Jakub, Wendy K. Nevala, Trace A. Christensen, Klara Noble-Orcutt, Zohar Sachs, Tina J. Hieken, Svetomir N. Markovic

**Affiliations:** ^1^Department of Immunology, Mayo Graduate School, Mayo Clinic, Rochester, MN, USA; ^2^Department of Surgery, Mayo Clinic, Rochester, MN, USA; ^3^Department of Oncology, Mayo Clinic, Rochester, MN, USA; ^4^Microscopy and Cell Analysis Core Facility, Mayo Clinic, Rochester, MN, USA; ^5^Division of Hematology, Oncology, and Transplantation, Department of Medicine, University of Minnesota, Minneapolis, MN, USA

**Keywords:** extracellular vesicles, dendritic cells, lymphatics, metastasis, tumor immunology

## Abstract

Evolution of melanoma from a primary tumor to widespread metastasis is crucially dependent on lymphatic spread. The mechanisms regulating the initial step in metastatic dissemination *via* regional lymph nodes remain largely unknown; however, evidence supporting the establishment of a pre-metastatic niche is evolving. We have previously described a dysfunctional immune profile including reduced expression of dendritic cell (DC) maturation markers in the first node draining from the primary tumor, the sentinel lymph node (SLN). Importantly, this phenotype is present prior to evidence of nodal metastasis. Herein, we evaluate melanoma-derived extracellular vesicles (EVs) as potential mediators of the premetastatic niche through cargo-specific polarization of DCs. DCs matured *in vitro* in the presence of melanoma EVs demonstrated significantly impaired expression of CD83 and CD86 as well as decreased expression of Th1 polarizing chemokines Flt3L and IL15 and migration chemokines MIP-1α and MIP-1β compared to liposome-treated DCs. Profiling of melanoma EV cargo identified shared proteomic and RNA signatures including S100A8 and S100A9 protein cargo, which *in vitro* compromised DC maturation similar to melanoma EVs. Early evidence demonstrates that similar EVs can be isolated from human afferent lymphatic fluid *ex vivo*. Taken together, here, we propose melanoma EV cargo as a mechanism by which DC maturation is compromised warranting further study to consider this as a potential mechanism enabled by the primary tumor to establish the premetastatic niche in tumor-draining SLNs of patients.

## Introduction

Regional lymph node metastasis remains among the most predictive prognostic markers for patients with solid tumors (1). Numerous studies in melanoma have demonstrated that the presence and number of metastatic lymph nodes directly correlate with decreased overall survival in patients presenting with clinical stages II (no lymph node metastases) and III (positive lymph node metastases) (2). As the treatment recommendations and prognosis is critically determined by lymph node status, lymphatic mapping and biopsy of the first tumor-draining lymph node or sentinel lymph node (SLN) has been adopted as the standard of care for clinical stage IB and II cutaneous melanoma (2). Its acceptance subsequently led investigators to better define the benefit of sentinel lymph node biopsy (SLNB) (3). To address this, the Multicenter Selective Lymphadenectomy Trial I was conducted as a randomized phase III clinical trial, which demonstrated in a cohort of 2001 patients with intermediate thickness melanoma, that SLNB prolongs disease-free survival for all patients and significantly improves melanoma-specific survival for node-positive patients (4). While the value of identifying SLN-positive disease has been evaluated clinically, the immunological changes underlying the tumor permissiveness of the SLN remains poorly characterized.

The epicenter of the adaptive immune response, the lymph node, anatomically localizes antigen-presenting dendritic cells (DCs) in proximity to antigen-specific lymphocytes to orchestrate effective responses between the detection and effector arms of immunity (5). In the tumor setting, tumor-draining SLNs also serve as the initial site of solid tumor (melanoma) metastases (6). In order for even microscopic metastases to harbor in seemingly immune-competent SLNs, changes to the lymphoid microenvironment are believed to be required (7). Initially described in Paget’s “seed and soil” hypothesis, our understanding of how tumor cells (the “seed”) prepare the microenvironment (the “soil”) for metastasis has relied predominantly on the role of tumor cells as drivers of metastasis (8). To define the lymphatic “soil” of regional lymph nodes in patients with melanoma, previous work in our laboratory utilized immunohistochemical (IHC) techniques to define the regional immune profile of SLNs with or without nodal metastasis compared to healthy control lymph nodes. Features including helper T cell polarization toward Th2, decreased expression of dendritic cell (DC) costimulatory marker CD86, and decreased CD8+ T cells differentiated melanoma SLNs from control lymph nodes obtained from patients undergoing prophylactic mastectomy (9). Notably, these changes in the SLN immune profile were independent of the presence of metastatic melanocytes. These initial findings in T cells were further evaluated in fresh lymphatic (SLN) tissue by flow cytometry techniques suggesting the existence of a premetastatic niche precedes clinically evident metastases in the SLN microenvironment (10); however, the mechanisms responsible for the effects on the monocytic lineage in the SLN remain unexplored. Taken together, increasing evidence suggests that while tumor cells actively contribute to the chronic repolarization of their metastatic microenvironments, the initial events responsible for permitting early (first metastatic cell) regional metastasis precedes even tumor cells.

Within the lymph node, DCs are the predominant antigen-presenting cells responsible for detecting and presenting tumor-associated antigens to lymphocytes (5). Matured from the monocyte lineage, DC maturation is tightly regulated by the requirement of three sequential signals including (1) MHC expression for antigen presentation (2); expression of costimulatory/maturation markers CD80 (B7-1), CD83, and CD86 (B7-2) that bind receptors on stimulated effector lymphocytes; and (3) cytokine production to modulate the ultimate nature of the effector immune response (11). Beyond these canonical signals, DC subsets can be further classified by differential expression of various receptors including DC1 and DC2 polarizing surface receptors CD11c and CD123, respectively (12). Taken together, these phenotypic markers in conjunction with the surrounding cytokine and chemokine milieu provide critical insight regarding the functions of DCs downstream.

Recently, extracellular vesicles (EVs) have emerged as mediators of a novel mechanism of intercellular communication (13). Secreted from nearly all cell types, membrane-bound EVs are composed of lipid, protein, and nucleic acid contents derived from the parent cell. Unlike soluble factors that are often limited in eliciting their effects in close proximity to their cell of origin, EVs have the unique capacity to transfer their contents to target cells across both paracrine and endocrine distances (14). Within the context of immune regulation, EVs have demonstrated various functions in modulating immune responses *in vitro* and *in vivo* (13). Originating from immune cells, EVs drive immune activation through direct antigen presentation to T and B cells (15, 16), indirect antigen presentation through antigen transfer to DCs (17), and activation of natural killer (NK) cells (18) and macrophages (19). In contrast, EVs originating from tumor cells have demonstrated immune inhibitory functions including decreased NK and CD8+ T cell cytotoxicity (20) and promotion of regulatory T cells (21) and myeloid-derived suppressor cells (MDSCs) (22). Recently, tumor-derived EVs have also demonstrated the capacity to inhibit DC maturation from the monocyte lineage (23); however, the mechanisms regulating this process have not been elucidated. In this study, we evaluate hypoxia-induced human melanoma-derived EVs for their role in regulating DC maturation in a cargo-dependent manner.

## Materials and Methods

### Patient Samples

Informed consent was obtained from all subjects participating in the following studies, following study approval by the Mayo Clinic Institutional Review Board.

#### Peripheral Blood

Peripheral blood from anonymous healthy donors was collected as clinical residual and peripheral blood mononuclear cells (PBMCs) isolated by Ficoll gradient.

#### Lymph Node Tissue

Fresh lymphatic tissue was collected as previously described (10). Briefly, lymph node specimens and proximal afferent lymphatic channels were obtained from patients with melanoma undergoing lymphatic mapping and SLNB as a standard of care (melanoma cohort). Control lymph nodes were obtained from women undergoing prophylactic mastectomy with no evidence of cancer (healthy control cohort). Following lymph node collection and initial pathology review, two non-contiguous 1-mm shavings were submitted for this research study, and the remainder of the frozen tissue was reviewed per routine clinical practice. Intact and clamped afferent lymphatic channels were dissected from the SLN following SLNB in the melanoma cohort. Research specimens were transported in RPMI media and lymph nodes processed into a single-cell suspension using Miltenyi gentleMACs Dissociator (Miltenyi Biotech, Bergisch Gladbach, Germany) according to the manufacturer’s instructions.

### CyTOF Analysis of SLNs

Briefly, single-cell suspensions of fresh SLN tissue was cultured overnight at 37°C in RPMI media (Corning) supplemented with 10% FBS (Millipore) and 1% penicillin, streptomycin, and glutamine (PSG) in the presence of 20 IU/mL IL-2 (Prometheus). Following incubation, cells were labeled with viability cisplatin (Cell-ID, Fluidigm) for dead cell exclusion and washed in MaxPar cell staining buffer (Fluidigm). Cells were subsequently fixed in 1.6% paraformaldehyde, blocked with anti-Fc receptor antibodies, and labeled with antibodies to cell surface markers including CD11a (142Nd), ICOS (143Nd), CD8a (146Nd), CD11c (147Sm), PDL1 (148Nd), HLA-DR/DP (150Nd), CD123 (151Eu), Tim-3 (153Eu), CD3 (154Sm), CD86 (156Gd), CCR7 (159Tb), CD14 (160Gd), CD80 (162Dy), Lag-3 (165Ho), CD25 (168 Tm), CTLA-4 (170Er), CD20 (171Yb), CD4 (174Yb), PD-1 (175Lu), CD56 (176Yb), and CD16 (209Bi). Following 30-min incubation with metal-conjugated antibodies, cells were washed and permeabilized in methanol prior to intracellular staining with T-bet (161Dy), Galectin-9 (163Dy), GATA3 (167Er), and Granzyme B (173Yb). After 30-min incubation, cells were again washed and labeled with intercalation solution (Iridium) for resolution of single-cell events. Stained cells were washed, resuspended in water containing calibration beads, and analyzed by CyTOF instrument (Fluidigm). High-dimensional single-cell analysis was conducted by viSNE analysis (Cytobank) sampling 25,000 cellular events/SLN using clustering channels CD3, CD4, CD8, CD20, CD56, CD14, and HLA-DR.

### Cell Lines

Human melanoma cell lines SKMEL28 (HTB-72), A375 (CRL-1619), and C32TG (CRL-1579), and normal, primary adult epidermal melanocytes (PCS-200-013) were purchased directly from American Type Culture Collection (ATCC), and all cell lines were passaged fewer than 10 times prior to storage to preserve authenticity. SKMEL28 and C32TG cells were cultured in Minimum Essential Media supplemented with 1% non-essential amino acids and A375 cells cultured in Dulbecco’s Modified Eagle Medium (Corning). All melanoma cell lines were additionally supplemented with 10% exosome-free FBS (Systems Bioscience) and 1% PSG (Corning) and cultured at 37°C and 5% CO_2_. Primary epidermal melanocytes were cultured with dermal cell basal medium supplemented with adult melanocyte growth kit according to the manufacturer’s instructions (ATCC). Routine screening of *Mycoplasma* contamination was conducted with MycoProbe Mycoplasma Detection Assay (R&D Systems) most recently in September 2016. All cell lines measured with negative OD values of <0.05.

### Hypoxic Cell Culture

For hypoxia induction, Petaka G3 low oxygen transfer flasks (Neuromics) were used to culture melanoma cell lines or primary melanocytes for 48–96 h at 37°C until >80% confluent.

### EV Isolation and Quantification

For EV quantification and coculture experiments, EVs were isolated from cell culture supernatant using Total Exosome Isolation Reagent (ThermoFisher) according to the manufacturer’s instructions (reagent isolation). For RNA characterization, recombinant protein experiments, and electron microscopy (EM) visualization, conditioned media or lymphatic fluid was prefiltered with a 0.8-µm Minisart NML syringe filter (Sartorius), and EVs were isolated on membrane-affinity spin columns by exoEasy maxi kit (Qiagen) according to the manufacturer’s instructions (column isolation). Unloaded liposomes (Sigma) were resuspended and sonicated for 30 s to be of similar size distribution and concentration for use as a negative control. To quantify total particle concentration and size distribution, nanoparticle tracking analysis (NTA) was performed with NanoSight NS300 (Malvern, UK). Vesicle preparations were diluted 1:100 (reagent isolation) or 1:1000 (column isolation) in HPLC-grade water and loaded into the sample chamber with sterile 1-mL syringe and visualized with NTA 2.3 software. Three video captures of 30 s each were taken of each vesicle preparation and particle concentration and size distribution calculated utilizing manual shutter and gain adjustments. Following analysis by the software, vesicle size was reported as mean particle size ± SEM and particle concentration as total concentration ± SEM.

### Immunogold Labeling and Negative Staining

Isolated EVs were concentrated by ultrafiltration on 100-K filters (Millipore), adhered to carbon-coated 200 mesh Cu grids, and labeled with CD63 antibody (BD Biosciences). Grids were incubated with secondary 10 nm gold-conjugated antibody with matching isotypes. Following a wash in 0.1 M phosphate buffer, pH 7.0, grids were fixed with 4% paraformaldehyde + 1% glutaraldehyde and negative stained with 1% phosphotungstic acid. Micrographs were acquired using a JEOL1400 Plus transmission electron microscope operating at 80 kV (Peabody, MA, USA).

### Transmission Electron Microscopy

Segments of lymphatic channel (2–4 mm in length) were fixed in 4% paraformaldehyde with 1% glutaraldehyde in phosphate-buffered saline, pH 7.2. Following fixation, channels were processed with the use of a laboratory microwave oven (Pelco BioWave 3450, Ted Pella, Inc., Redding, CA, USA). Briefly, tissue was secondarily fixed with 1% osmium tetroxide and 2% uranyl acetate, dehydrated through a graded ethanol series, and embedded into Embed 812 resin. Following a 24-h polymerization at 60°C, 0.1-µm ultrathin sections were poststained with lead citrate. Micrographs were acquired using a JEOL1400 Plus transmission electron microscope operating at 80 kV (Peabody, MA, USA).

### *In Vitro* DC Culture

CD14+ monocytes were negatively selected (Miltenyi Biotech) from the PBMCs of healthy human donors. Monocytes were cultured with 10 ng/mL GM-CSF (Genzyme) and 1 ng/mL IL-4 (R&D Systems) for 5 days and matured with 100 ng/mL CD40L (R&D Systems) for 2 days. Differentiating monocytes were cocultured with SKMEL28-derived EVs or liposomes (1 × 10^10^ particles/mL) from day 0, or recombinant proteins S100A8 (Abcam), S100A9 (ThermoFischer), Annexin A1 (R&D Systems), and/or ICAM1 (R&D Systems) at 10 µg/mL from day 5.

### Flow Cytometry and ImageStream

Dendritic cell surface markers were assessed by flow cytometry on Day 7 using surface markers CD14, CD11c, CD123, HLA-DR, HLA-ABC, CD83, AND CD86 (BD Pharmingen). Statistical significance was determined using two-tailed *t*-tests (*p* < 0.05). For imaging flow cytometry, prior to coculture with DCs, melanoma-derived EVs were isolated and fluorescently stained with general lipid membrane marker PKH67 (Sigma) according to the manufacturer’s instructions. Briefly, 2 µL of PKH was diluted in 0.5 mL Diluent C and then mixed with EV preparation for 4 min. Following the incubation, 1 mL complete culture media was added, and EVs were washed and concentrated over 100-K ultrafilters (Millipore). After 7-day coculture, DCs were analyzed using ImageStream MKII imaging flow cytometer (Amnis) and analyzed using Ideas 6.2 software (Amnis).

### Cytokine and Chemokine Multiplex

Cytokine and chemokine protein concentrations were measured in cell culture supernatants using the MagPlex Milliplex human 38-analyte multiplex panel (Millipore) per the manufacturer’s instructions and as described (24). The analytes measured include EGF, FGF-2, eotaxin, TGF-α, G-CSF, Flt3L, GM-CSF, fractalkine, IFNα2, IFN-γ, GRO, IL-10, MCP-3, IL-12p40, MCD, IL-12p70, IL-13, IL-15, sCD40L, IL-17A, IL-1RA, IL-1α, IL-9, IL-1β, IL-2, IL-3, IL-4, IL-5, IL-6, IL-7, IL-8, IP-10, MCP-1, MIP-1α, MIP-1β, TNF-α, TNF-β, and VEGF. Assay was read by Luminex xPONENT technology (Luminex, Austin, TX, USA). Analyte concentrations were quantified using a standard curve with a range of 1.6 and 5,000 pg/mL, using Milliplex Analyst software (VigeneTech, Carlisle, MA, USA). All samples were run in duplicate and averaged to determine concentration.

### EV Proteomics

Extracellular vesicles were cultured under hypoxic and reduced serum conditions. For hypoxia induction, Petaka G3 low oxygen transfer flasks (Neuromics) were used to culture melanoma cell lines for 48 h at 37°C until >80% confluent. Purified EVs were then lysed, and protein content was measured by BCA assay and run on 4–20% TGX Ready gel (Bo-Rad). Protein bands were visualized by silver stain technique, according to the manufacturer’s instructions (ThermoFisher). Silver-stained gel sections were prepared for MS analysis as previously described (25). Briefly, gel fractions were destained in 15 mM potassium ferricyanide:50 mM sodium dithionite:H_2_O solution, reduced, and alkylated with 50 mM TCEP and 20 mM idoacetamide for 2 h in the dark. Acetonitrile-dehydrated gel sections were digested in 5 ng/µL trypsin overnight at 37°C. Peptides were then extracted in 2% TFA followed by acetonitrile, and both extract solutions combined, dried, and stored at −80°C. LC-MS/MS analysis was conducted on either a Thermo Scientific Orbitrap Elite Hybrid Mass Spectrometer or QExactivePlus (Thermo Fisher Scientific, Bremen, Germany) both coupled to a Thermo Ultimate 3000 RSLCnano HPLC systems, using handpacked Magic C18 3 μm: 25 cm × 75-μm columns at ambient temperatures and Magic C18 5 μm trap at a flow rate of 325 nL/min. The Orbitrap Elite mass spectrometer experiment was set to perform a FT full scan from 340–1,500 *m/z* with resolution set at 120,000 (at 400 *m/z*), followed by linear ion trap rapid CID MS/MS scans on the top 25 ions. The QExactivePlus experiment performed a FT full scan from 340–1,500 *m/z* with resolution at 70,000 and HCD on the 20 ions at 17,500. Tandem mass spectra were extracted by MSConvert, and all MS/MS samples were analyzed using Mascot (Matrix Science, London, UK; version 2.4.0) and X! Tandem (The GPM, http://thegpm.org; version X! Tandem Sledgehammer (2013.09.01.1)) set up to search the Swissprot all species database (Nov2014, 1,093,672 entries) with reverse decoy sequences. Both searches assumed the digestion enzyme trypsin and a parent ion tolerance of 10.0 PPM with fragment ion mass tolerance of 0.60 Da for the Elite or 0.02 Da for the QXactive Plus raw files. Oxidation of methionine and the iodoacetamide derivative of cysteine were specified as variable modifications. Scaffold (version Scaffold_4.6.2, Proteome Software Inc., Portland, OR, USA) was used to validate MS/MS-based peptide and protein identifications. Peptide identifications were accepted if they could be established at greater than 95.0% probability by the Scaffold Local FDR algorithm or the Peptide Prophet algorithm (26) and contained at least two identified peptides. Protein probabilities were assigned by the Protein Prophet algorithm (27). The mass spectrometry proteomics data have been deposited to the ProteomeXchange Consortium *via* the PRIDE partner repository with the data set identifier PXD005539. Protein identifications matched across all three melanoma cell line EV preparations (SKMEL28, C32TG, A375) were reported, as well as EV specific proteins and intracellular proteins present in non-EV cellular compartments for EV characterization.

### Real-time RT-PCR

Isolated EVs were eluted from column with QIAzol lysis buffer and RNA isolated by exoRNeasy kit (Qiagen) following the manufacturer’s instructions. RNA content was quantified by spectrophotometer DS-11 (DeNovix), and equal concentrations of each EV RNA preparation were then converted to cDNA with RT^2^ First Strand Kit (Qiagen). The cDNA was mixed with RT^2^ SYBR Green Mastermix before loading in a 96-well array containing genes specific to the Th1 and Th2 pathways (Qiagen). The reactions were read by real-time cycler (ABI ViiA7), and fold regulation was calculated for each gene using RT^2^ Profiler PCR Array Data Analysis software.

## Results

**Figure 1:**
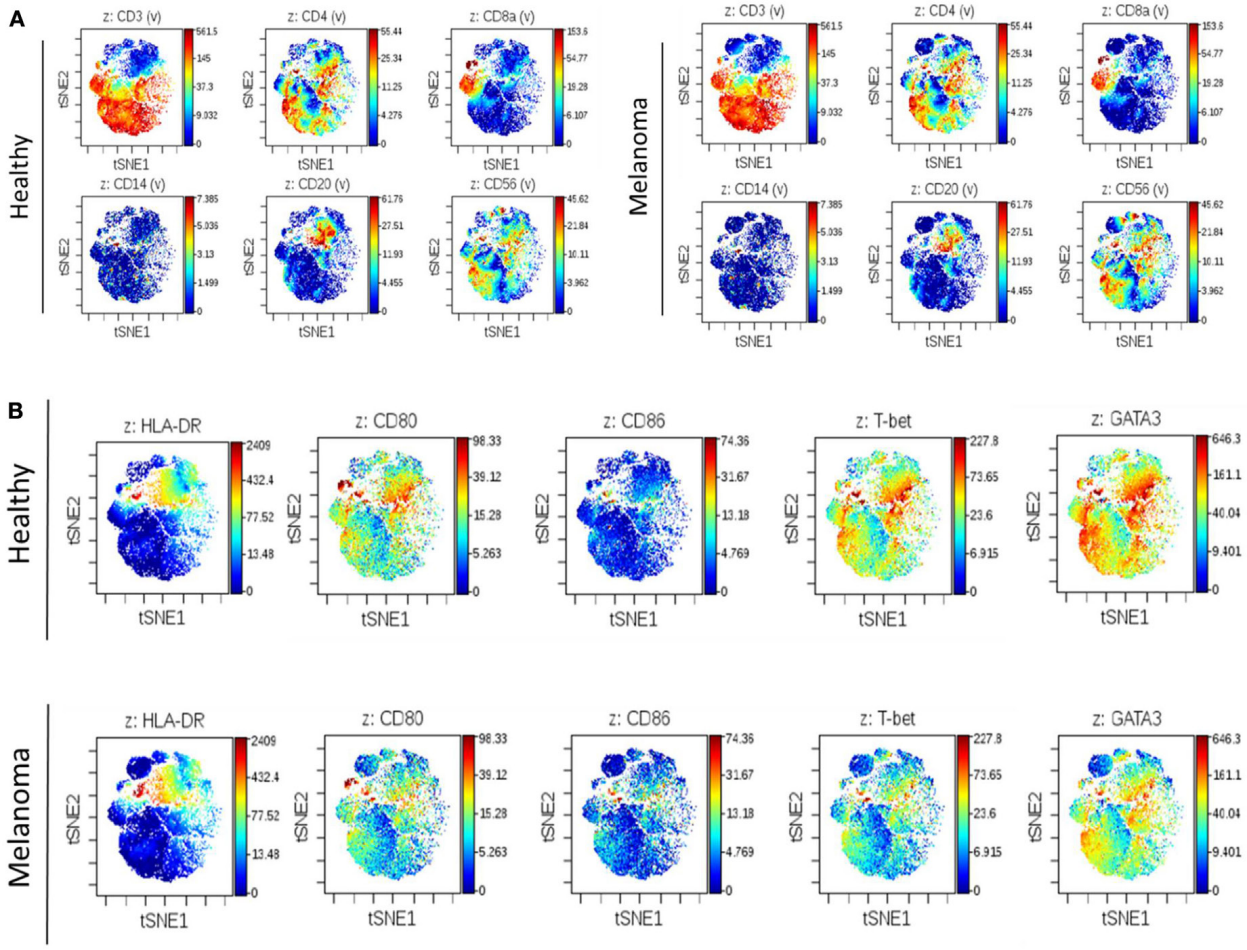
**Illustration of altered regional immune profile signatures preceding clinical evidence of nodal metastasis**. Mass cytometry of 25 analytes was performed on single-cell suspensions of premetastatic SLN and healthy lymph node. Illustrative images of viSNE analysis plots of broad immune cell surface markers in melanoma and control SLNs **(A)**. Global differences in surface markers HLA-DR, CD80, and CD86 and transcription factors T-bet and GATA3 were similarly compared. Decreased CD80 and T-bet expression was observed in premetastatic SLN compared to control lymph node **(B)**. On viSNE plot each cell is depicted as a single dot on the plot, and marker expression is indicated by color intensity.

To complement our previous IHC work assessing changes in the immune cell composition of human SLNs prior to metastasis, we performed deep phenotyping of multiple immune cell parameters using mass cytometry on freshly isolated SLN tissue. In this pilot analysis, fresh lymph node tissue was obtained from a patient with melanoma undergoing SLNB (clinically negative for metastatic disease) compared to a healthy lymph node obtained from a patient undergoing prophylactic mastectomy procedure. Utilizing a 25 analyte panel, differences in a variety of immune cell subsets could be assessed simultaneously. No differences were observed in the overall distribution of broad immune cell subsets including T cells (CD4+ and CD8+), B cells (CD20+), monocytes (CD14+), and NK cells (CD56+) when comparing premetastatic melanoma to control lymph nodes (Figure 1A). However, an overall decrease in DC costimulatory marker CD80 and Th1 polarizing transcription factor T-bet expression was observed in the premetastatic SLN compared to the healthy node (Figure 1B). This illustration coincides with the earlier IHC studies, which showed a statistically significant decrease in DC maturation marker CD86 and a decrease in CD8+ T cells and an overall shift from Th1 to Th2 polarization (9). Additional studies by Grotz et al. further characterized the loss of CD8+ T cells in the SLN prior to metastasis (10); however, mechanisms exploring the compromised DC maturation phenotype were not elucidated. Therefore, we chose to develop an *in vitro* model in this study to evaluate the effects of melanoma-derived EVs on DC maturation.

**Figure 2:**
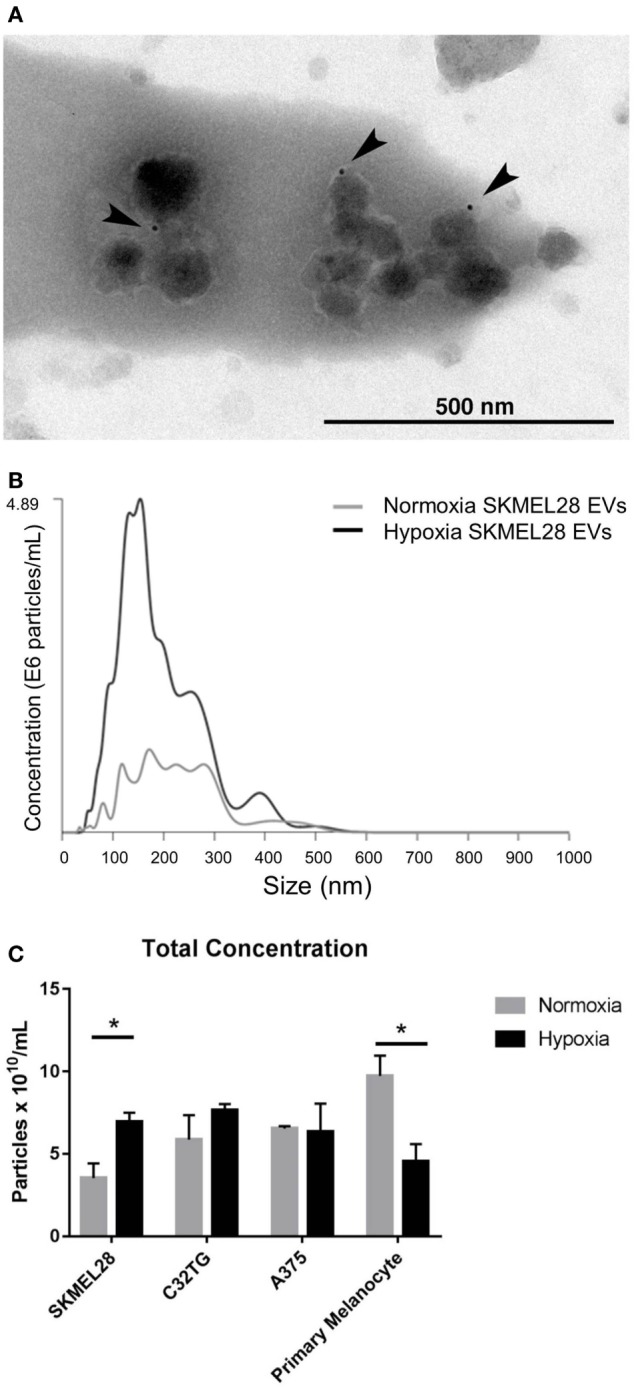
**Hypoxia increases extracellular vesicles (EVs) production in metastatic melanoma cell line SKMEL28 not primary melanocytes**. Melanoma cell lines and adult primary melanocytes were cultured under hypoxia or normoxia conditions for 48 h. Morphology and CD63 vesicle marker expression of SKMEL28 EVs was confirmed by immune electron microscopy **(A)** and vesicle size distribution and particle concentration measured by nanoparticle tracking analysis (Nanosight) [**(B)**, representative image]. Total concentration of normoxic (gray) or hypoxic (black) vesicles isolated from three melanoma cell lines and healthy primary melanocytes was quantified by Nanosight following three replicate 30-s video captures of three EV preparations **(C)** (*n* = 3 independent experiments). Statistical significance was determined by two-tailed *t*-test analysis (*p < 0.05).

As melanoma penetrates the layers of the dermis to gain access to the draining lymphatics, a hypoxic core develops in which selective pressures encourage evolution of transformed cells and induce secretion of angiogenic factors responsible for initiating neovascularization (28). Thus, we sought to evaluate the effects of hypoxic stress on EV production in human malignant melanocytes *in vitro*. Following hypoxic culture, EVs (including both exosome and microvesicle populations) were isolated from the cultured supernatant of three melanoma cell lines. To rigorously define the heterogenous EV population isolated, the vesicles were characterized by proteomic assesment, size distribution, morphology and immunogold staining by EM. To ensure EV purity from other non-EV cellular compartments, transmembrane, lipid-bound, and cytosolic proteins enriched in EVs were quantified by mass spectrometry (Table S1 in Supplementary Material). Proteomic profiles of each of the EV preparations showed enrichment for six or more EV-associated proteins and had a single histone (SKMEL28, A375) or no intracellular proteins (C32TG) associated with non-EV compartments, demonstrating high EV purity. To qualitatively assess morphology and expression of vesicle membrane marker CD63, EVs isolated from SKMEL28 melanoma cell line were visualized by immuno-electron microscopy (Figure 2A).

To characterize the composition of exosome and microvesicle populations within the melanoma cell line EVs isolated, vesicle size distribution and total concentration were first quantified and compared to EVs isolated from adult primary melanocytes under normoxic and hypoxic conditions (Figures 2B,C). By nanoparticle tracking analysis (NTA), average vesicle size of hypoxic EVs did not vary significantly from EVs cultured under normal conditions for any of the EV sources analyzed (Table S2 in Supplementary Material); however, vesicle production was found to be significantly increased in the melanoma cell line SKMEL28 under hypoxic compared to normal culture conditions (6.94 × 10^10^ vs. 3.53 × 10^10^ particles/mL, *p* = 0.03). Inversely, EV production was significantly decreased in primary non-malignant melanocytes cultured under hypoxic stress compared to normal conditions (4.55 × 10^10^ vs. 9.73 × 10^10^ particles/mL, *p* = 0.03) (Figure 2C). This data suggests melanoma EVs can be generated under hypoxic conditions *in vitro* amenable to the hypoxic core observed in invading primary tumors *in vivo*.

**Figure 3:**
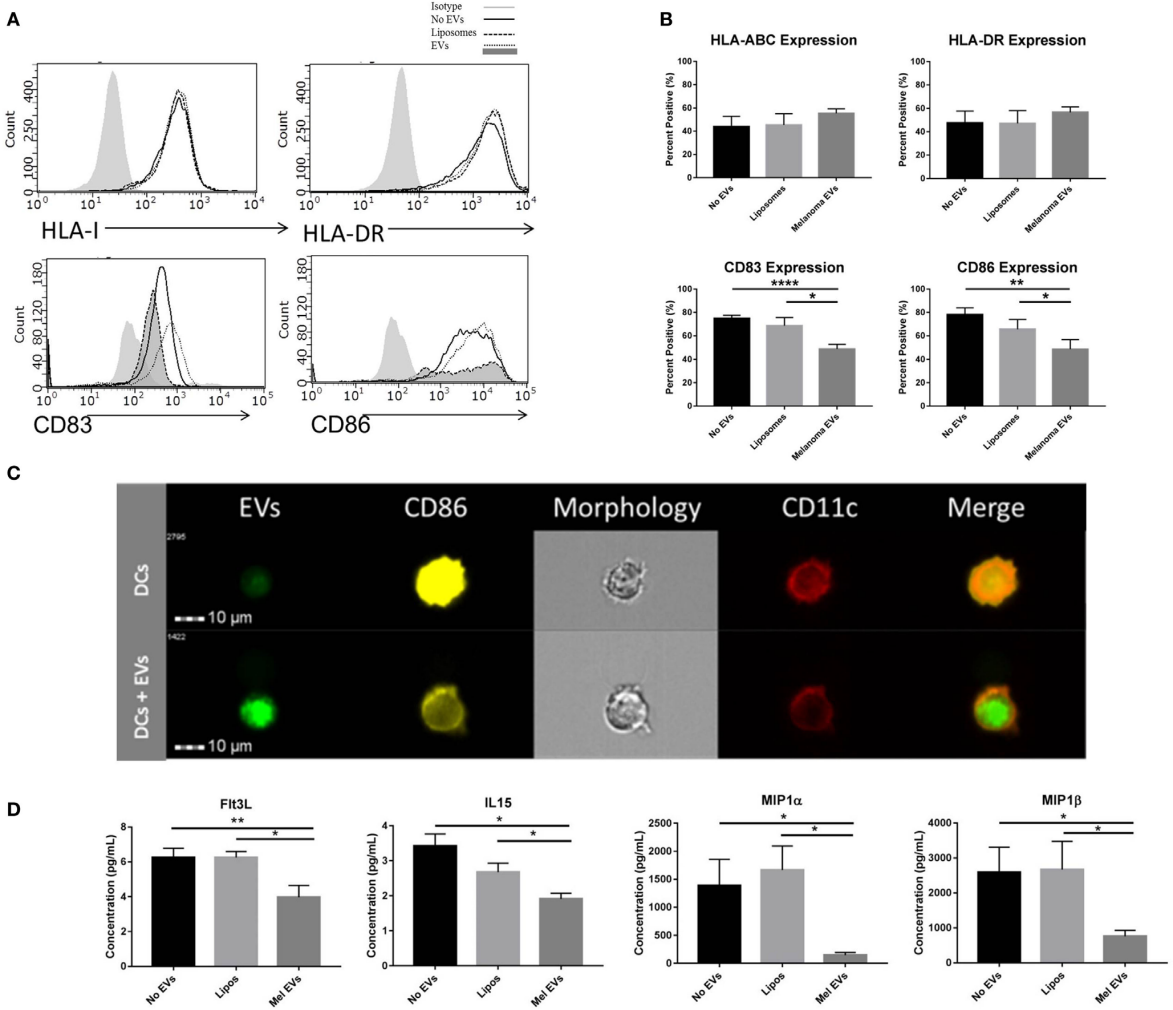
**Melanoma-derived extracellular vesicles (EVs) suppress dendritic cell (DC) maturation *in vitro***. CD14+ human monocytes were matured to DC fate with CD40L in the absence or presence of SKMEL28 melanoma EVs, empty liposomes, or PBS. On day 7, phenotyping by flow cytometry evaluated DC expression of MHC molecules (HLA Class I, HLA-DR) and costimulatory molecules (CD83 and CD86) by histogram or the percent positive cells (mean ± SEM) (*n* = 8) **(A,B)**. Representative image of reduced CD86 expression by imaging flow cytometry in DCs cocultured with fluorescently labeled melanoma EVs (*n* = 3) **(C)**. Multiplex results of four analytes significantly decreased (Flt3L, IL-15, MIP-1α, and MIP-1β) in the presence of melanoma EVs compared to liposome-treated control (*n* = 5) **(D)**. Cytokine and chemokine concentrations were reported in picogram per milliliter (mean ± SEM). Statistical significance was determined by two-tailed *t*-test analysis (*p < 0.05, **p < 0.01, *p* < 0.001). [EV-treated DCs are denoted by dotted line and dark gray shading on histograms for CD83 and CD86 expression in panel **(A)**.]

Next, we sought to determine whether melanoma-derived EVs had the propensity to regulate DC maturation and the surrounding cytokine and chemokine milieu. Negatively isolated CD14+ monocytes from PBMCs of healthy human donors were cocultured to the DC fate *in vitro* with CD40L in the presence or absence of SKMEL28-derived EVs or empty liposomes. To evaluate the sequential maturation of DCs, expression of Class I and II MHC complexes, maturation markers CD83 and CD86 and cytokine and chemokine profiles were defined. Phenotyping by flow cytometry, expression of Class I and Class II MHC molecules did not vary in response to EV treatment suggesting that initial antigen presentation is not likely affected by melanoma-derived EVs. Interestingly, expression of costimulatory markers CD83 and CD86 were significantly decreased in DCs treated with melanoma-derived EVs compared to either liposome-treated DCs or DCs alone (Figures 3A,B). Inhibition of costimulation was confirmed by imaging flow cytometry whereby fluorescently labeled melanoma EVs colocalized with DCs and correlated with decreased CD86 expression compared to DCs matured in the presence of CD40L alone (Figure 3C). Overall, these data demonstrate that melanoma EVs negatively regulate human monocyte maturation to the DC fate at the costimulatory stage of DC differentiation.

To define the surrounding milieu of the *in vitro* EV-treated DCs, culture supernatants were collected on day 7 following monocyte-derived DC maturation, and quantification of 38 cytokines and chemokines was performed by multiplex analysis. From this panel, four chemokines were found to be differentially regulated by DCs cultured in the presence of melanoma-derived EVs compared to a liposome-treated controls (Figure 3D). Decreased levels of Th1-polarizing Flt3L and IL-15 chemokines and decreased levels of DC-recruiting chemokines (29), MIP-1α and MIP-1β, were observed in the conditioned media of monocytes matured with melanoma EVs, suggesting in addition to compromising maturation, EV-conditioned DCs may elicit functional effects on immune cells downstream and modulate DC migration.

To elucidate the cargo present within melanoma-derived EVs, protein composition of the melanoma EVs was interrogated by mass spectrometry. Protein lysates of EVs yielded a shared proteomic signature of 21 proteins among all three melanoma cell lines assessed [Table 1(A)]. Strikingly, 11 of these 21 proteins had previously been identified as playing a functional role in DC maturation including inhibition of costimulation expression mediated by S100A8 (30), S100A9 (31), Annexin A1 (32), Annexin A2 (33), and ICAM1 (34) [Table 1(B)].

**Table 1:** **Profiling of melanoma-derived extracellular vesicle (EV) protein cargo: Venn diagram summarizing the number of unique proteins identified in EVs isolated from 3 melanoma cell lines of which 21 proteins were shared among all 3 EV preparations (A); total peptide count of the 21 proteins identified in the shared signature and associated roles in dendritic cell (DC) maturation (B)**.

**(A)**	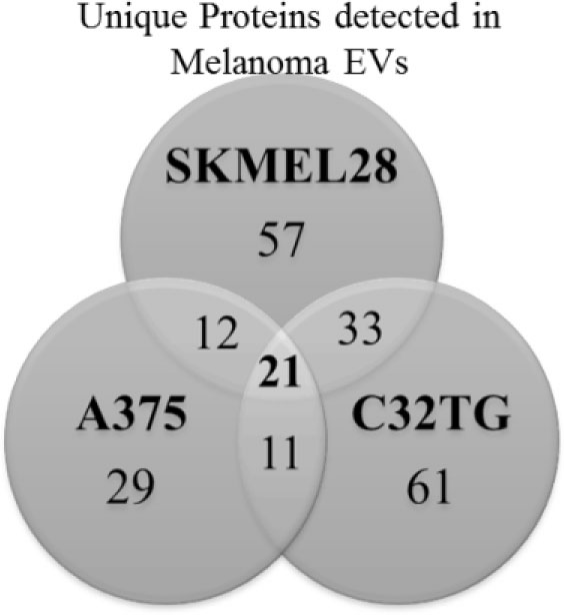	**(B)**	**Protein name**	**Peptide count**			**Functional role in dendritic cell (DC) maturation**	**Reference**
				**SKMEL28**	**C32TG**	**A375**		
		
			S100A8	5	5	4	Inhibitory	(30)
			S100A9	3	5	3	Inhibitory	(31)
			Annexin A1	4	9	12	Inhibitory	(32)
			Annexin A2	23	26	10	Inhibitory	(33)
			Heat shock cognate protein 71 kDa	17	12	19	Activating	(35)
			Fibronectin	10	14	26	Activating	(36)
			Laminin subunit gamma 1	15	16	9	Activating	(36)
			Prolactin-inducible protein	2	6	3	Activating	(37)
			Procollagen-lysine	4	17	9	Activating	(38)
			ICAM1	6	4	4	Inhibitory/activating	(34, 39)
			Annexin A5	11	21	3	–	
			Chloride intracellular channel protein	2	4	3	–	
			Lysozyme C	3	4	3	–	
			Alpha enolase	2	22	15	–	
			Glyceraldehyde-3-phosphate dehydrogenase	3	17	6	–	
			Glucose 6 phosphate isomerase	2	12	5	–	
			Glutathione S transferase	2	5	4	–	
			Fructose bisphosphate aldolase	9	16	7	–	
			Gelsolin	3	6	7	–	
			Tenascin	52	11	16	–	
			14-3-3 Protein zeta delta	6	12	4	–	

In addition to protein cargo, EVs have demonstrated the capacity to transport nucleic acids. To evaluate if melanoma-derived EVs were enriched for immune-modulating mRNAs, 84 genes involved in generating downstream T helper cell-, Th1-, and Th2-specific responses were measured by qRT-PCR. Following amplification, 14 genes were found to be differentially increased in melanoma EVs relative to primary melanocyte EVs, each with defined T cell-polarizing effects (Table 2). These findings suggest melanoma-derived EVs are selectively enriched for cargo with the potential to modulate T cell polarization and DC differentiation.

**Table 2:** **Defining melanoma-derived EV RNA cargo**.

Gene name	Gene abbreviation	Fold change (relative to primary melanocyte EV)
Secreted phosphoprotein 1	*SPP1*	6.29
Signal transducer and activator of transcription 1	*STAT1*	6.17
Toll-like receptor 4	*TLR4*	6.06
CD40 ligand	*CD40LG*	5.24
Proto-oncogene c-Maf	*MAF*	4.89
Toll-like receptor 6	*TLR6*	4.47
Polycomb group ring finger 2	*PCGF2*	3.96
Solute carrier family 11 member 1	*SLC11A1*	3.74
Growth factor independent 1	*GFI1*	3.64
Janus kinase 1	*JAK1*	3.61
Interleukin 9	*IL9*	3.56
Vascular endothelial growth factor A	*VEGFA*	3.34
CCAAT/enhancer binding protein beta	*CEBPB*	3.17
Interleukin 24	*IL24*	3.17

*Fold regulation of significant (detection threshold ≥3-fold change) genes enriched by quantitative RT-PCR in SKMEL28 EVs relative to primary melanocyte EVs (*n* = 3 independent experiments)*.

**Figure 4:**
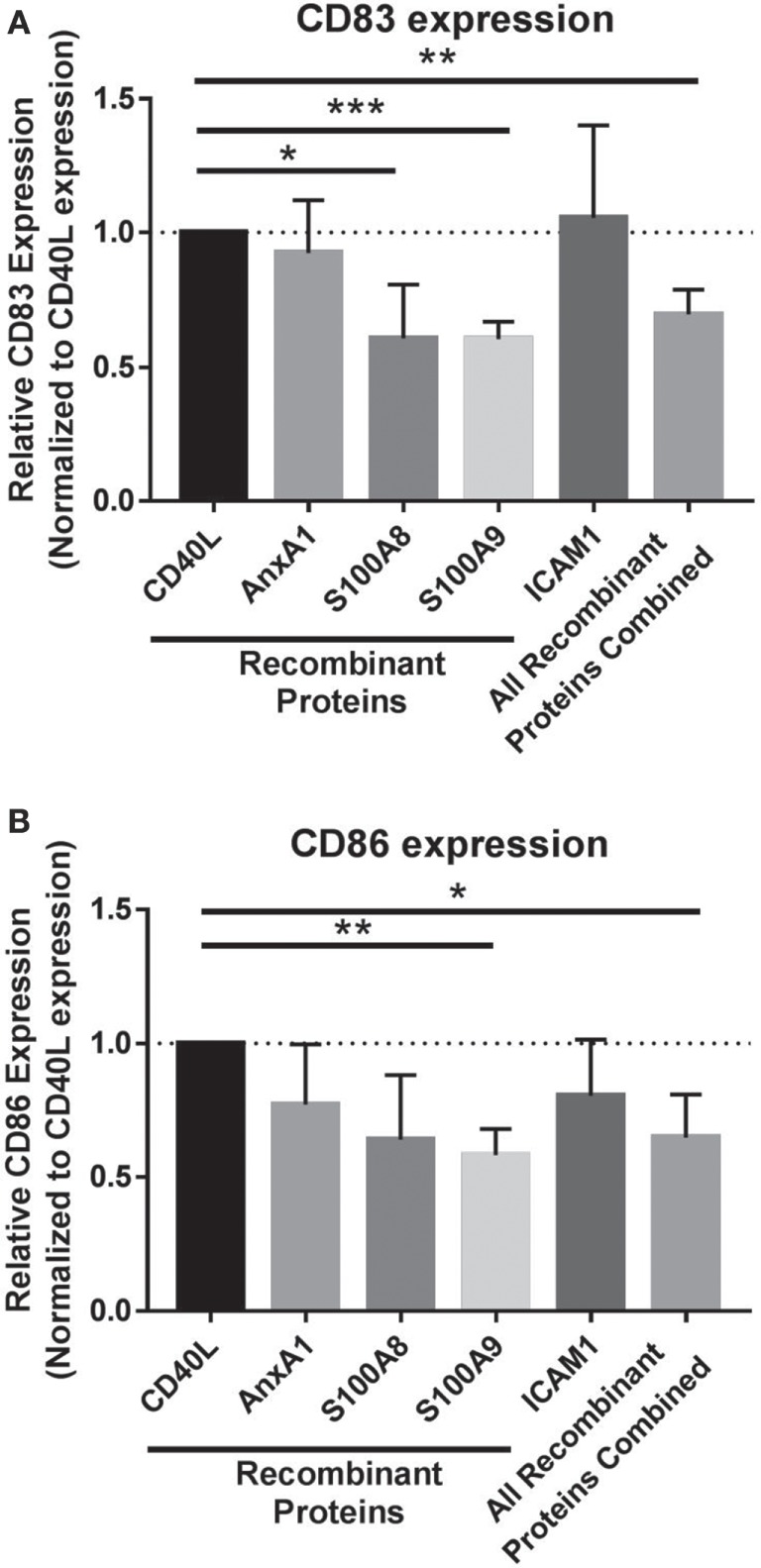
**Extracellular vesicles (EVs) cargo works synergistically to drive compromised dendritic cell (DC) phenotype**. Maturation of DCs was evaluated by CD83 expression **(A)** and CD86 expression **(B)** on day 7 following coculture with recombinant proteins specific to EV protein cargo or all of the recombinant proteins administered in combination (all of the recombinant proteins combined). Statistical significance was determined by two-tailed *t*-test analysis (*p < 0.05, **p < 0.01, ***p < 0.001).

To isolate the effects of individual and combined EV cargo in regulating this process, DC maturation was conducted in the presence of the EV-associated proteins with identified inhibitory functions on DCs. *In vitro*, monocytes were matured to DCs with CD40L and coadministered recombinant proteins on day 5 specific to S100A8, S100A9, ICAM1, and AnnexinA1 to determine whether DC maturation could be modulated by these proteins alone or in combination. Individually, S100A8 and S100A9 proteins as well as all four inhibitory EV proteins combined significantly decreased CD83 expression in DCs compared to DCs matured with CD40L alone (Figure 4A). DC maturation was also assessed by CD86 expression, whereby DCs matured in the presence of all proteins combined resulted in decreased CD86 expression while S100A9 was the only protein able to decrease CD86 expression individually (Figure 4B). Taken together, these data suggest melanoma EV cargo, including established chronic inflammatory mediators S100A8 and S100A9 have the capacity to drive EV inhibitory effects on DC maturation.

**Figure 5:**
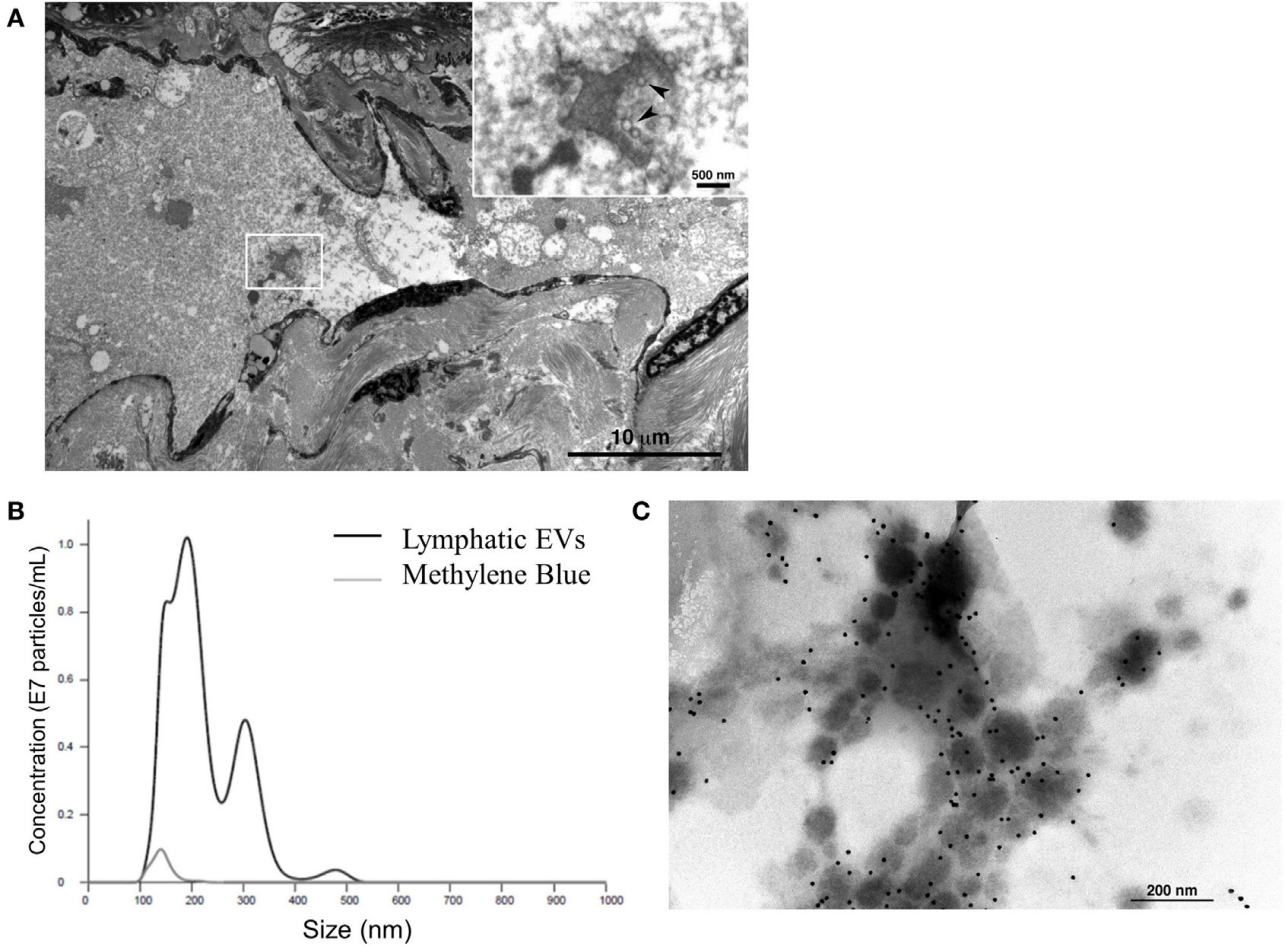
**Characterization of novel human lymphatic extracellular vesicles (EVs)**. Electron micrographs confirm vesicle structures (inset, denoted by arrows) in the lumen of human afferent lymphatic channels **(A)** EVs isolated from lymphatic fluid entering SLN were visualized and quantified (black line) compared to methylene blue, channel tracer dye (gray line) alone by nanoparticle tracking analysis **(B)**. Isolated lymphatic EVs express vesicle marker CD63 by immunogold labeling **(C)**.

Finally, to consider the implications of this *in vitro* model in humans, we assessed afferent lymphatic channels draining into the identified, premetastatic SLN of patients for evidence of trafficking EVs. Our initial analysis of afferent lymph fluid identified a novel source of lymphatic EVs. Size distribution of human lymphatic EVs was determined by NTA with an average vesicle size of 197.8 nm (Figure 5A), and electron micrographs collectively confirm the presence of vesicular structures within the lumen of the channel (Figure 5B) and strong CD63 expression on isolated lymphatic EVs (Figure 5C). To our knowledge, this is the first published evidence of EVs in human lymphatics.

## Discussion

In this study, we examined the function of melanoma-derived EVs as proficient signaling complexes capable of compromising DC maturation and regulating cytokine and chemokines in the surrounding milieu. Characterization of the proteomic and genomic cargo within melanoma EVs specifically identified four proteins with known immune-modulating functions, which in combination decreased DC maturation *in vitro*. DC maturation was similarly assessed in the presence of individual EV cargo proteins demonstrating that S100A8 decreases expression of the costimulatory marker CD83 while coculture of DCs with S100A9 resulted in decreased expression of both CD83 and CD86 suggesting that EV cargo is responsible for mediating the immature DC phenotype. Finally, evidence of EVs within human afferent lymphatic channels provides the first correlative evidence in support of EVs trafficking from the primary tumor to draining SLNs in patients.

Serving dual functions by orchestrating antitumor immune responses and fostering the first site of metastasis, the SLN comprises a unique immunological environment to study early stages of melanoma progression. Angiogenic factors including VEGF, TGF-β, and TNF-α have been previously shown to play an active role in priming the lymphoid microenvironment for melanoma migration; however, the events responsible for inducing secretion of these factors are largely unknown (40). In patients, we previously demonstrated by IHC and flow cytometry studies that changes in basic immune cell subsets and polarization within the SLN precede evidence of nodal metastasis including decreased CD8+ T cells, increased monocytes, and decreased expression of DC maturation marker CD86 (9, 10). To appreciate a more comprehensive immune phenotype of SLNs at the resolution of single cells, our current study examined freshly isolated SLN immune profiles by mass cytometry (Figure 1). At the earliest stages of melanoma, a SLN clinically negative for metastatic spread compared to a healthy lymph node illustrated a conserved phenotype in agreement with these earlier studies suggesting that decreased expression of T-bet and decreased DC costimulatory marker CD80 are early steps in regional lymph node remodeling.

Historically, intercellular communication mechanisms responsible for mediating immune responses have focused on cell-to-cell and receptor–ligand mediated interactions (41) as well as soluble cytokines, chemokines, and angiogenic factors (42). Recently, EVs have emerged as novel mediators of intercellular communication composed of both exosome (30–100 nm) and microvesicle (100 nm–1 μm) subpopulations. In addition to their size, exosomes are defined by their endosomal origin and microvesicles by their budding from the plasma membrane; however, exclusive cargo markers to differentiate these vesicles based on their downstream functions on the recipient cell are less clear. Here, we assessed the functional effects of melanoma EVs on DCs and therefore chose to evaluate a heterogeneous population of both exosomes and microvesicles as we hypothesized both populations are abundant *in vivo* and may work in tandem to modulate DC maturation.

Transporting signals from their parent cell to a target cell through cargo, numerous studies have extensively profiled the proteomic and genomic cargo present in EVs obtained from various cell sources (43). From these studies, diverse immune regulatory functions of EVs have been defined including antigen presentation, NK cell activity and T cell activation (13). In the cancer setting, increased tumor-derived EVs circulating in plasma have been proposed as potential biomarkers for disease progression (44). *In vivo*, Peinado et al. provided the first evidence to suggest that melanoma exosomes have prometastatic potential by educating bone marrow-derived progenitor cells in mice (45), while few studies have considered the mechanistic role of tumor-derived EV cargo in driving functional effects on non-malignant cells. The stroma and target site of spread is likely just as critical to survival of the malignant cells as the metastatic cells themselves. In this study, we identified melanoma-derived EVs containing cargo enriched for immune regulatory proteins including S100A8, S100A9, Annexin A1, and ICAM1 (Table 1) and further demonstrated that all of the proteins as well as S100A8 or S100A9 individually have the functional capacity to compromise DC maturation (Figure 4).

First described as potent antimicrobials secreted by neutrophils, S100A8 and S100A9 proteins form a stable heterodimer, calprotectin, with established immunogenic functions (46). Members of the larger S100 calcium-binding protein family, S100A8 and S100A9, regulate epithelial cell differentiation with increased expression of these proteins observed in inflammatory conditions and advanced stages of skin cancer (47). S100A8 and S100A9, individually or as a heterodimeric complex, signal through cell surface receptors TLR4 and RAGE resulting in Myd88-mediated NF-kB activation downstream (47). *In vivo* murine studies conducted by Cheng et al. demonstrated that overexpression of S100A9 inhibited DC and macrophage differentiation and promoted MDSCs (31). In this study, we evaluated EV protein cargo for their capacity to regulate CD83 and CD86 expression in DCs and observed that DCs cultured in the presence of S100A8 and S100A9 had impaired maturation. Interestingly, protein ligands S100A8 and S100A9 and cognate receptor TLR4 were all identified in melanoma EV cargo. Future work is focused on elucidating if conserved signaling pathways are present within EV cargo and may further inform mechanisms responsible for modulating target cell fates.

To our knowledge, this is the first study examining the direct effects of melanoma-derived EV cargo on compromised DC maturation, a DC phenotype observed in pre-metastatic human SLNs. In addition, our preliminary evidence suggests that similar EVs are present within human lymphatics and ongoing studies are exploring the immune-modulatory functions of these novel EVs on immune cell subsets. Taken together, this work suggests melanoma EVs, and their associated cargo serve as modulators of DC maturation and cytokine/chemokine production *in vitro* and should be further characterized in human lymphatics to assess a potential functional role for EVs in harboring a premetastatic SLN niche permissive for future tumor dissemination.

## Ethics Statement

This study was carried out in accordance with the recommendations of the Mayo Clinic Institutional Review Board and Biospecimens committee with written informed consent obtained from all subjects in accordance with the Declaration of Helsinki. The protocol was approved by the Mayo Clinic Institutional Review Board.

## Author Contributions

All authors provided critical insight in regards to study design, data collection, analysis and interpretation, and critical evaluation of article preparation prior to publication. RM conducted experiments, monitored data collection, and drafted the manuscript. JJ served as the surgical lead for SLN and lymphatic channel collection and JJ and TH implemented the SLN study in the clinic providing surgical expertise and patient specimens. ZS initiated collaborative work at UMN utilizing CyTOF technology, providing key insights into study design and data interpretation. KN-O developed assay methodologies and collected data for CyTOF analysis. TC developed EM techniques to visualize melanoma and human lymphatic EVs by *ex vivo* and *in situ* approaches. WN supervised laboratory experiments, and WN and SM provided valuable mentorship, assisted with study design, data interpretation, and manuscript revisions.

## Conflict of Interest Statement

The authors declare that the research was conducted in the absence of any commercial or financial relationships that could be construed as a potential conflict of interest.

## Abbreviations

EVs, extracellular vesicles; SLN, sentinel lymph node; DC, dendritic cell.
