# Supplementary figures and images for: Human Melanoma-Derived Extracellular Vesicles Regulate Dendritic Cell Maturation
Source: Front Immunol. 2017 Mar 29;8:358. doi: 10.3389/fimmu.2017.00358 (PMC5372822; doi:10.3389/fimmu.2017.00358)

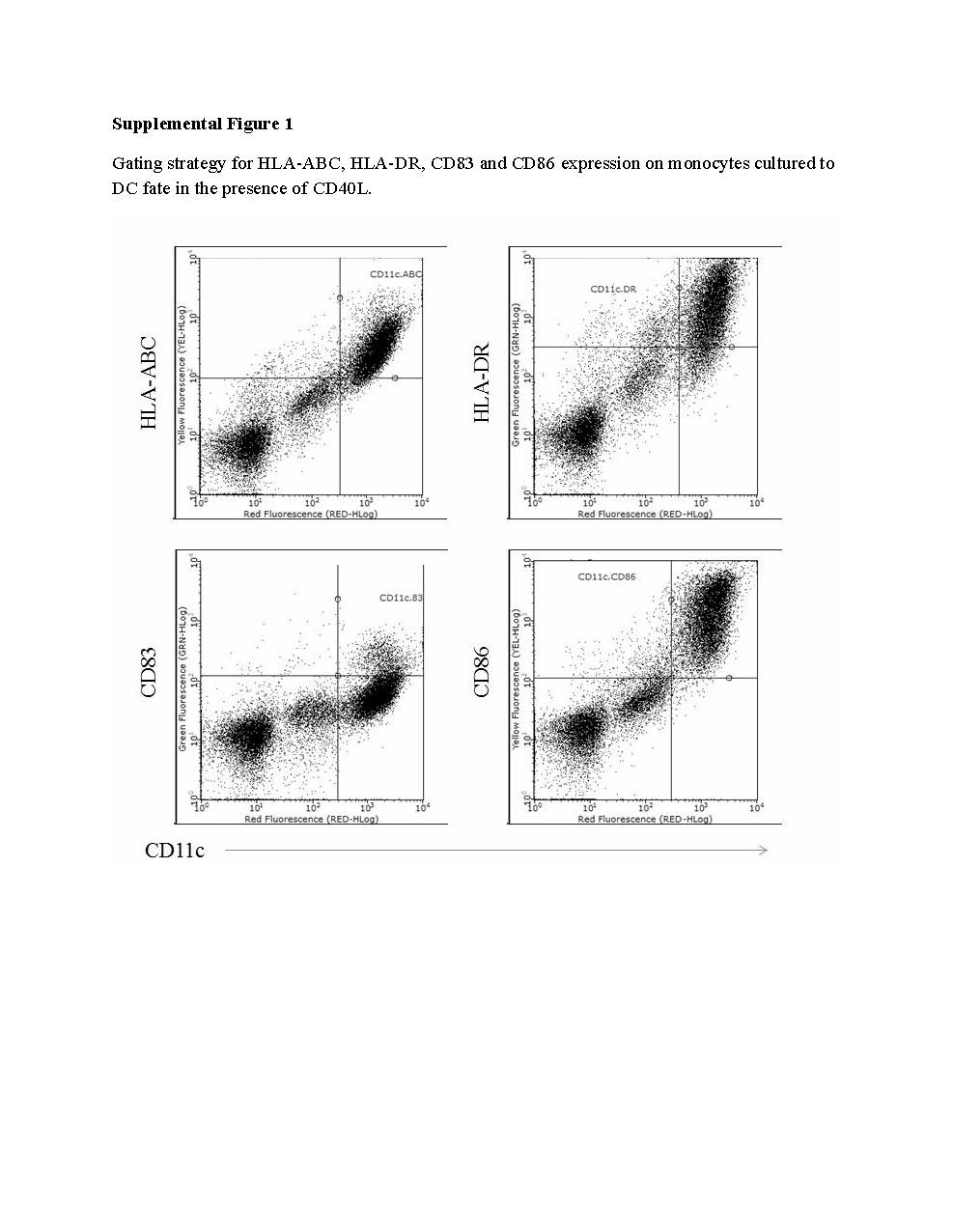

Supplement: Supplementary file 1 [file image_1.jpeg]

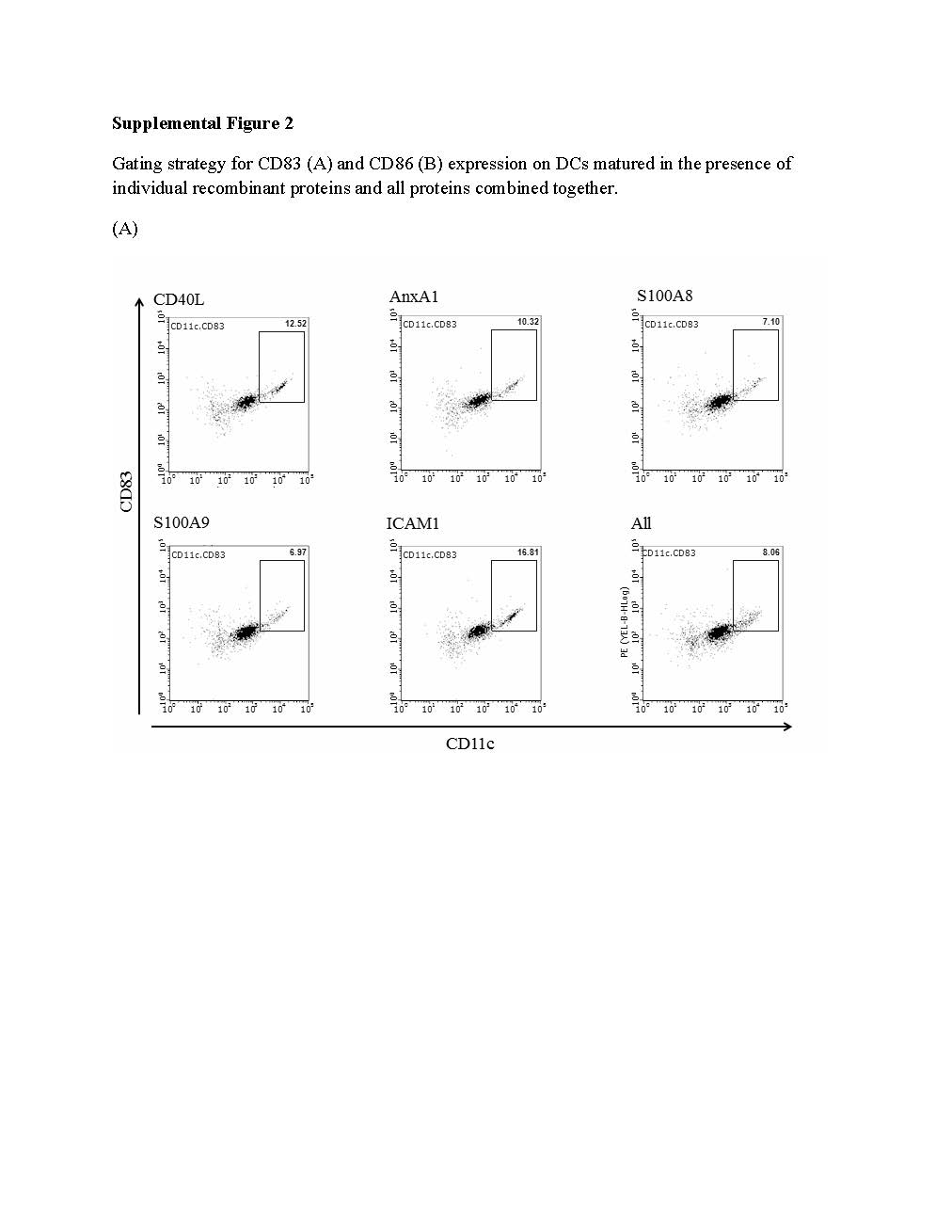

Supplement: Supplementary file 2 [file image_2.jpeg]

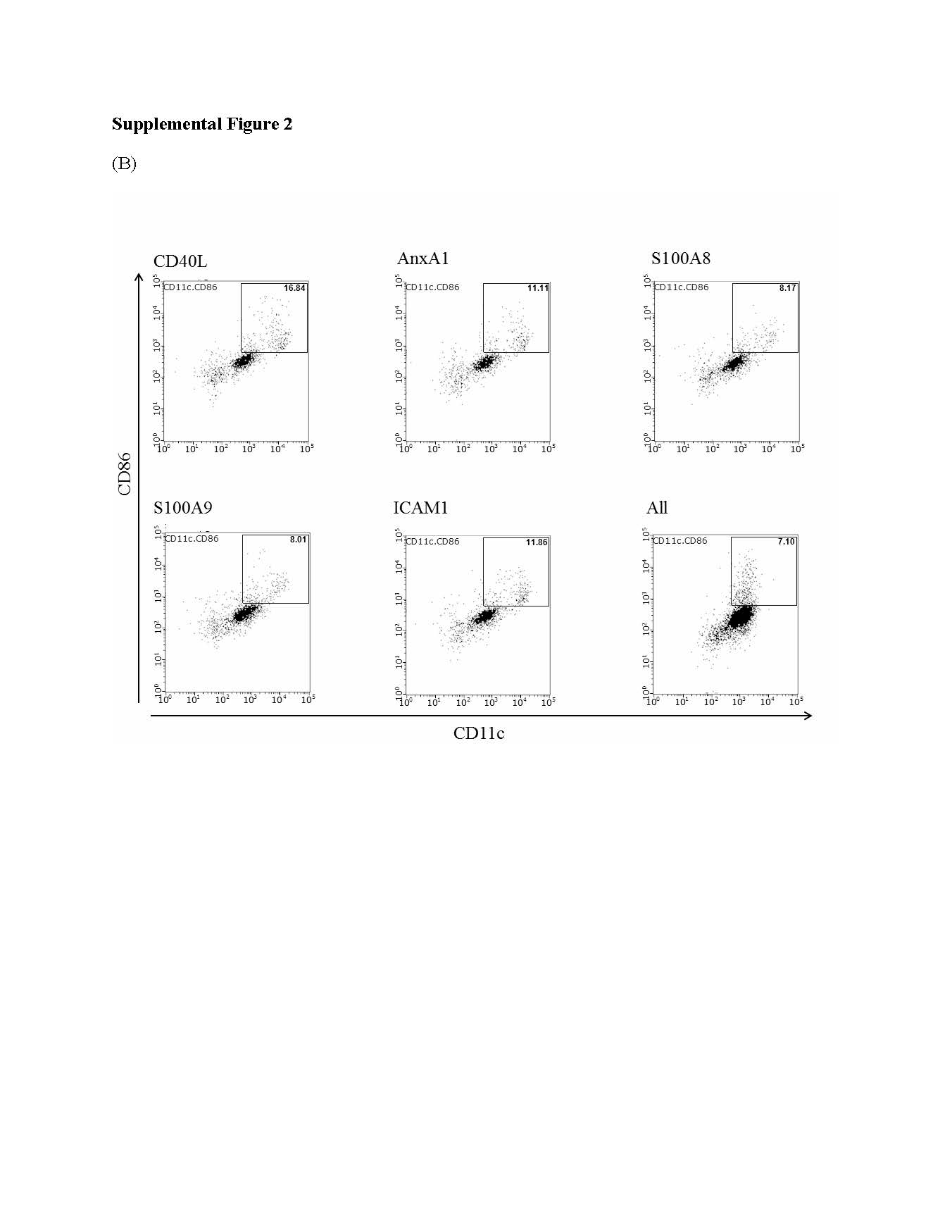

Supplement: Supplementary file 3 [file image_3.jpeg]
