# Supplementary material for: Human Melanoma-Derived Extracellular Vesicles Regulate Dendritic Cell Maturation
Source: Front Immunol. 2017 Mar 29;8:358. doi: 10.3389/fimmu.2017.00358 (PMC5372822; doi:10.3389/fimmu.2017.00358)
Supplement: Supplementary file 4 [file table_1.docx]

**Supplemental Table 1**

Characterization of melanoma cell line EVs by mass spectrometry. Quantification of peptide counts specific to transmembrane/lipid bound extracellular proteins and cytosolic proteins [as described in Journal of Extracellular Vesicles 2014, 3: 26913] in EV preparations from melanoma cell lines SKMEL28, C32TG, and A375. In addition to the presence of EV-associated proteins, we also assessed peptide counts of intracellular proteins associated with non-EV cellular compartments including the endoplasmic reticulum (HSP90B1, CANX), Golgi (GM130), mitochondria (CYC1), nucleus (histones) and argonuate/RISC complex. In all of the cell line EV preparations, only histone H1.4 and histone 2b were detectable in SKMEL28 and A375 EV preparations respectively, each with a peptide count of 3, suggesting high EV purity.
