# Supplementary material for: Human Melanoma-Derived Extracellular Vesicles Regulate Dendritic Cell Maturation
Source: Front Immunol. 2017 Mar 29;8:358. doi: 10.3389/fimmu.2017.00358 (PMC5372822; doi:10.3389/fimmu.2017.00358)
Supplement: Supplementary file 5 [file table_2.docx]

**Supplemental Table 2**

Quantification of mean particle size and total concentration for melanoma-derived and healthy melanocyte-derived EVs cultured under normoxic and hypoxic conditions utilizing nanoparticle tracking analysis.
